# Rose Bengal Immobilization in and Release from Calcium Alginate Gels for Singlet-Oxygen-Generating Photosensitizing Systems

**DOI:** 10.3390/gels12080741

**Published:** 2026-08-19

**Authors:** Alexander Kopylov, Anastasiya Cherkasova, Ilya Shershnev, Valentin Bekeshev, Nadezhda Aksenova, Victoriya Timofeeva, Anastasiya Akovantseva, Tatyana Zarkhina, Valeriya Kardumyan, Peter Timashev, Anna Solovieva

**Affiliations:** 1 N.N. Semenov Federal Research Center for Chemical Physics Russian Academy of Sciences, 4 Kosygina Street, Moscow 119991, Russia; via_cetra@mail.ru (A.K.); anastasiya-cherk@mail.ru (A.C.); bvg495@yandex.ru (V.B.); naksenova@mail.ru (N.A.); vik.timofeeva@gmail.com (V.T.); akovantseva-a@yandex.ru (A.A.); zarkhina@mail.ru (T.Z.); valerysik@yandex.ru (V.K.); timashev.peter@gmail.com (P.T.); 2 Institute of Fine Chemical Technologies, Russian Technological University, 86 Vernadskogo Prospekt, Moscow 119571, Russia; 3 Institute for Regenerative Medicine, Sechenov University, 8 Trubetskaya Street, Moscow 119991, Russia

**Keywords:** rose bengal, calcium alginate, xerogels, alcogels, aerogels, singlet oxygen, photogeneration

## Abstract

Calcium alginate (CaA) xerogels and aerogels were evaluated as carriers for Rose Bengal (RB) in singlet-oxygen-generating systems intended for photodynamic applications. Hybrid CaA/polyvinylpyrrolidone (PVP) matrices were also prepared. Nitrogen sorption showed that the CaA xerogels(A) were mesoporous, with a specific surface area of S_BET_ ~225 m^2^/g and a mean pore diameter of approximately 9.3 nm; the corresponding S_BET_ values were approximately 300 m^2^/g for the aerogels and below 1 m^2^/g for the xerogels. RB introduced during xerogel formation from water was retained in the matrix and was not released into phosphate-buffered saline (PBS, pH 7.2). By contrast, RB loaded from isopropanol was released from the xerogels(A) and aerogels, with the fastest release observed for CaA aerogels. RB incorporated into xerogels(A) and aerogels retained singlet-oxygen-generating activity. In air, the highest luminescence response was observed for RB immobilized in hybrid CaA/PVP aerogel films containing approximately 10 wt% PVP. These findings suggest that such systems hold promise for applications in photodynamic therapy of superficial skin injuries. Xerogels(A) are also attractive carrier candidates because they avoid supercritical drying and, for the CaA xerogel(A) studied here, released 50% of the loaded RB within approximately 5 min.

## 1. Introduction

The dianionic xanthene dye Rose Bengal (RB) is one of the most frequently used photosensitizers (PSs) for generating singlet oxygen, ^1^O_2_, in in vitro and in vivo processes (quantum yield in water, $\Phi_{\Delta }=0.76$) [1]. The halogenated xanthene ring in the RB structure accounts for its properties as a photosensitizer [2]. Owing to its good water solubility, high $\Phi_{\Delta }$, and long-lived triplet excited state (τ_1/2_ 0.1–0.3 ms), RB is a convenient agent for photodynamic therapy (PDT) in the treatment of oncological diseases and localized bacterial infections [3]. RB also possesses sonophotochemical properties that enable its use in sonodynamic therapy for eliminating tumors and bacteria, as well as in dermatological and cosmetic applications [4,5]. The photosensitizing properties of RB in the generation of ^1^O_2_ have been well studied, and it is often used as a standard when determining $\Phi_{\Delta }$ for newly synthesized photosensitizers [6]. RB is relatively inexpensive, biocompatible, and readily available, but it is used primarily as a dye because its photosensitizing properties have not undergone the appropriate clinical trials [7].

The limited clinical use of RB as a photosensitizer is associated with its relatively unstable biopharmaceutical profile. In some cases, the dye is deactivated or cleared from the affected area fairly rapidly (half-life ~30 min [8]). To improve the stability of the photosensitizing properties of Rose Bengal, it is generally chemically modified or immobilized on biocompatible carriers. Immobilization provides controlled release and uniform distribution of RB within the affected area [6]. In particular, covalently bound hydrophobic RB derivatives have been shown to penetrate cell membranes more readily. However, such penetration is not cell-selective, which increases dye accumulation in nontarget cells and thereby increases adverse effects [9]. Materials of various types, including nanoscale materials, are used as carriers for RB immobilization. Inorganic carriers include metal oxides (titanium, gadolinium, and iron oxides), metals (gold and silver), and silicon [10,11]. Organic carriers based on natural polymers (chitosan, proteins, and peptides) and synthetic polymers (polyesters of hydroxycarboxylic acids) are also employed [10,12].

In this work, photosensitizing systems were prepared using Rose Bengal immobilized in solid calcium alginate (CaA) gels (xerogels and aerogels). We previously developed polymeric photosensitizing systems based on methylene blue incorporated into crosslinked calcium alginate gels. Calcium alginate is used as a carrier for bioactive molecules in drug-delivery systems because of the polysaccharide’s ability to form a stable, water-insoluble, gel-like material under mild aqueous conditions upon the addition of multivalent cations [13]. The present study therefore examined the feasibility of forming polymeric photosensitizing systems from anionic RB and polyanionic CaA under conditions in which the dye is retained as an electrically neutral species. The principal objective was to establish how gel type, porosity, surface structure, and RB–matrix interactions affect RB release into buffer and singlet-oxygen-generation activity.

The cationic dye methylene blue was localized in the alginate matrix primarily as a bound counterion at the carboxyl groups of crosslinked calcium alginate. This conclusion is supported by its release into buffer solutions and the absence of leaching into water, indicating that the dye could be released via an ion-exchange mechanism [13,14]. For the anionic dye Rose Bengal, two distinct modes of immobilization in the polyanionic calcium alginate matrix are proposed. When the dye was introduced from an aqueous solution and the resulting hydrogel was air-dried, Rose Bengal was immobilized in the resulting xerogel, presumably near calcium cations, accompanied by aggregation of the dye molecules. In this state, the dye was not leached into either water or buffer solution. However, when the hydrogels were treated with isopropanol, Rose Bengal, likely due to its higher solubility in alcohol compared to water, could be retained in the matrix through donor-acceptor interactions with macromolecular fragments of alginate. With this binding method, the dye could be released from the alginate matrix into both water and buffer solutions. This demonstrated for the first time the versatility of the alginate matrix for immobilizing both cationic and anionic photoactive compounds. Preliminary experiments showed that, at the same dye loading in calcium alginate, rose bengal dye released into solution was more active in generating singlet oxygen than methylene blue.

## 2. Results and Discussion

### 2.1. Structure of the Solid Gels

#### 2.1.1. Chemical-Structure Features of Xerogels and Aerogels

##### IR Spectroscopy Data

**Figure 1:**
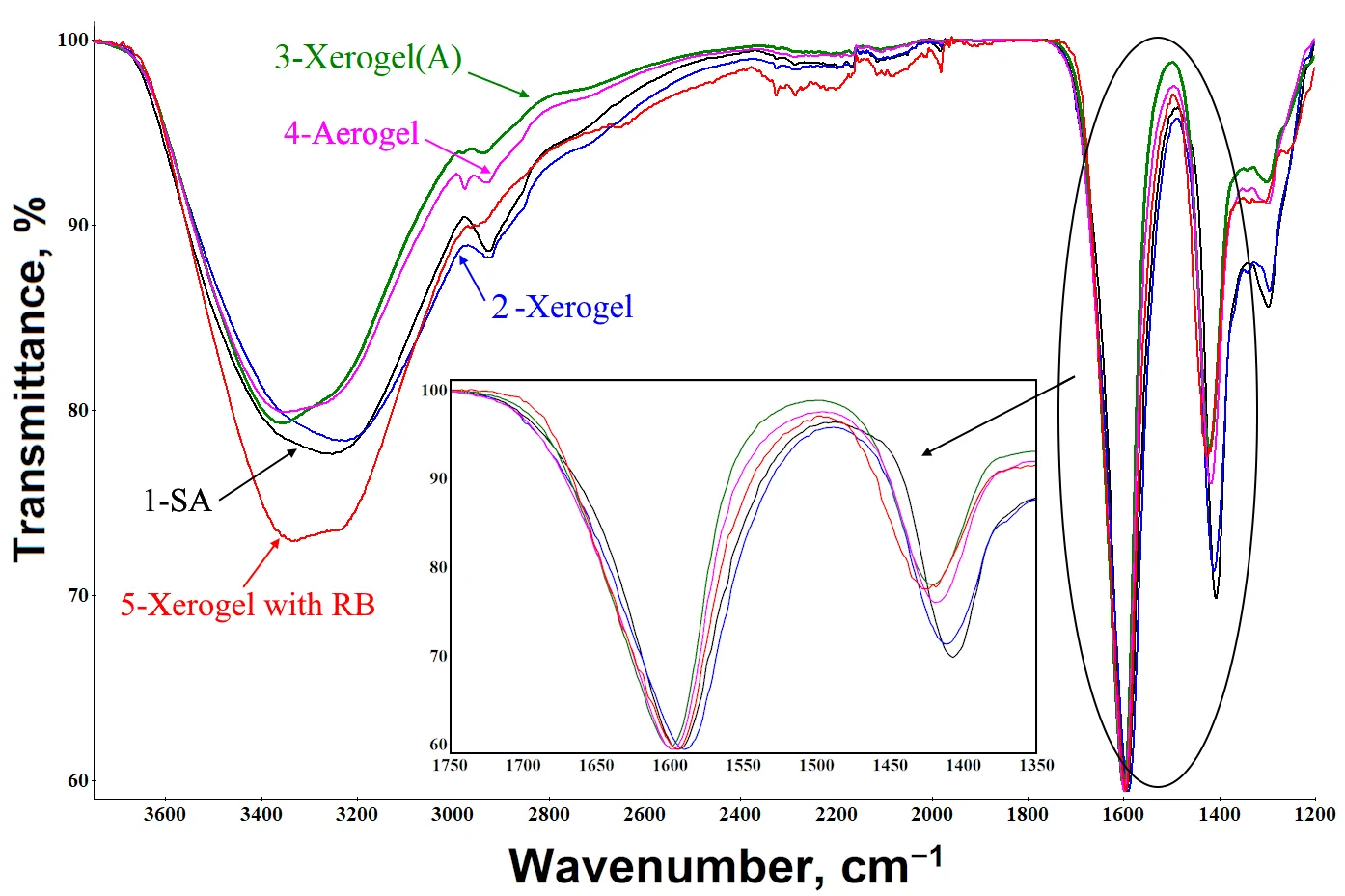
IRspectra of sodium alginate film (1); calcium alginate xerogel (2), xerogel(A) (3), and aerogel (4); and a calcium alginate xerogel containing Rose Bengal (5), over the wavenumber range 4000–1200 cm^−1^, inset—wavenumber range 1350–1750 cm^−1^.

Figure 1 presents the IR spectra of the initial sodium alginate—SA (1), xerogel (2), xerogel(A) (3), and aerogel (4).

The IR spectra of all alginate gels contain a broad band at 3400–3300 cm^−1^, assigned to the stretching vibrations of hydroxyl groups. These OH groups are constituents of the carbohydrate rings in the polysaccharide chains and participate in hydrogen bonding. OH groups may also be present in molecules of bound and free water. The gel spectra are also characterized by low-intensity bands near 2900 cm^−1^ assigned to the stretching vibrations of methyl and methylene groups (−CH_3_ and −CH_2_^−^). In addition, the spectra contain two peaks corresponding to the asymmetric and symmetric stretching vibrations of carboxylate groups (−COO^−^), at 1595–1590 cm^−1^ (high intensity) and 1410–1407 cm^−1^ (medium intensity). Several bands in the 1100–1000 cm^−1^ range are attributable to vibrations of the glycosidic bonds in the polysaccharide (C–O–C stretching vibrations) [15,16]. On going from sodium alginate (Figure 1, curve 1) to calcium alginate xerogel (curve 2), the band at 3400–3300 cm^−1^ becomes somewhat less intense, indicating the expected decrease in unbound-water content in the xerogel relative to the SA polymer film. This is supported by the markedly lower intensity of this band for the calcium alginate xerogels(A) and aerogels (Figure 1, curves 3 and 4). Two forms of bound water remain in the crosslinked alginate gel: hydration water, which forms strong hydrogen bonds with the hydroxyl and carboxyl groups of the polysaccharide, and less strongly bound structural water, which forms solvation shells around the macromolecules and crosslinking cations—in this case, calcium [17]. Figure 1 also shows that the carboxyl-group peaks in the CaA xerogels and aerogels (asymmetric and symmetric vibrations at 1595–1590 and 1410–1407 cm^−1^; curves 2, 3, and 4 in Figure 1) are shifted slightly toward higher wavenumbers relative to the SA spectrum. The carboxylate bands of the xerogels(A) and aerogels are also less intense than those in the SA spectrum. These differences may be associated both with dehydration of the COO^−^ groups during crosslinking and with distortion of the COO^−^ · Ca^2+^ bonds during formation of the cellular structure of the crosslinked alginate chains [15,16].

Incorporating RB into calcium alginate xerogels decreases the intensity of the 1417–1407 cm^−1^ band to the level observed for the “neat” xerogels(A) and aerogels. This may indicate that in the “filled” xerogel, the ratio of asymmetric to symmetric vibrations of carboxyl groups increases and that additional binding sites influence the mobility of COO^−^ groups. It can be assumed that the dye is incorporated near the polarized COO^−^⋯Ca^2+^ bonds. In addition, Figure 1 shows that upon dye incorporation, the carboxyl groups’ asymmetric-vibration band shifts from 1590 cm^−1^ for the calcium alginate xerogel to 1595 cm^−1^. This also indicates a change in the microenvironment of the COO^−^ groups.

It may be this interaction between RB anions and Ca^2+^ cations that prevents the release of the dye from calcium alginate xerogels.

##### DTA Data

The results of differential thermal analyses support the conclusions drawn from the IR spectra of the CaA xerogels and aerogels.

**Figure 2:**
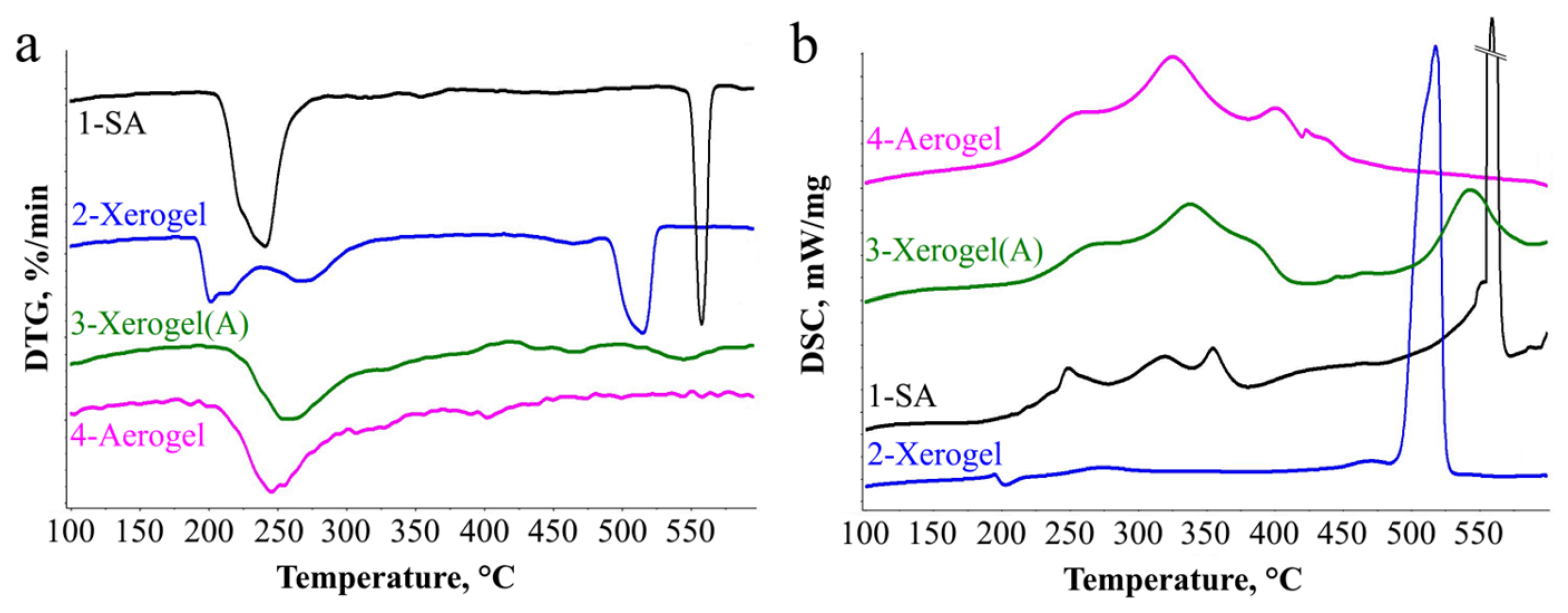
DTG (**a**) and DSC (**b**) curves for the initial SA (1) and calcium alginate xerogel (2), xerogel(A) (3), and aerogel (4).

The DTG and heat-release curves in Figure 2a,b show that all gel types undergo degradation by the same mechanism, indicating that their chemical structures are similar. As noted above, the xerogel evidently contains structural “bound water,” detected near 200 °C (Figure 2b, curve 2). Such bound water may affect the rearrangement of the xerogel’s supramolecular structure during degradation and the subsequent degradation behavior of the xerogel over the temperature range 220–500 °C. During crosslinking, bound water acts as a plasticizer: it coordinates around calcium ions and helps them become firmly fixed between two guluronic-acid units [18,19]. Importantly, bound water is not detected in the xerogels(A), aerogels, or initial sodium alginate (Figure 2, curves 1, 3, and 4). The presence of bound water may help RB anions become firmly fixed near calcium ions and prevent them from being washed out of the xerogel matrix.

#### 2.1.2. Spatial Structure of the Gels

**Figure 3:**
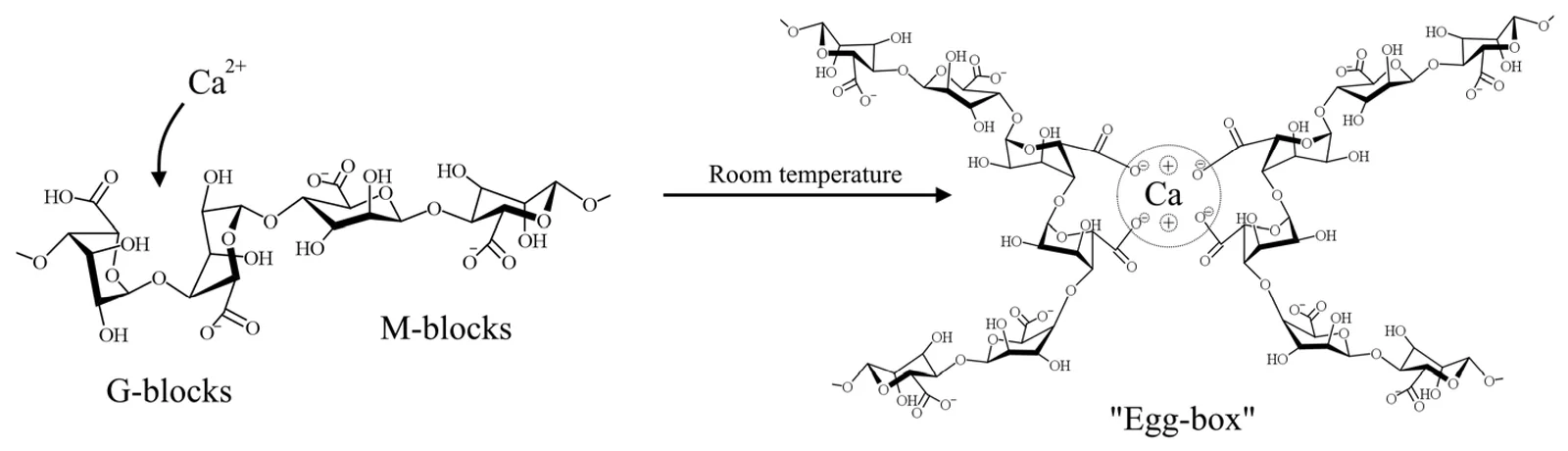
Schematic representation of the egg-box structure formed by interactions between the carboxylate ions of alginate guluronate units and Ca^2+^ ions.

The supramolecular structure of alginate forms when the polyanion is crosslinked by salts of multivalent metals. This ionic crosslinking produces a three-dimensional network, primarily through interactions between the carboxyl groups of guluronate units and multivalent cations, leading to the conformation known as the “egg-box” structure (Figure 3) [20,21].

The strength of the electrostatic interaction between metal cations used to crosslink alginate and the carboxyl groups of its macromolecules depends on the charge and nature of the metal: trivalent cations > Pb^2+^ > Cu^2+^ > Cd^2+^ > Ba^2+^ > Sr^2+^ > Ca^2+^ [22]. Although Ca^2+^ does not exhibit the strongest interaction, calcium salts are the most widely used agents for producing crosslinked alginate gels [23]. This choice may be attributable both to the relatively dense structure of calcium alginate gels and to the compatibility of calcium with the human body, particularly given its role as a principal component of the skeletal system and a regulator of various physiological processes.

It is important to emphasize that, according to X-ray diffraction data, alginate macromolecules associate with preformed dimers during the formation of egg-box structures. It can therefore be assumed that nearly half of the carboxyl groups do not participate in the formation of Ca^2+^ · −2(−OOC^−^) bonds [24]. Consequently, during subsequent interdimer association, the spaces between dimers may contain sodium or hydrogen ions that neutralize the excess charge of the carboxyl groups. Metal ions are believed to be capable of penetrating these interdimer-association regions even in the case of calcium, which, as noted above, interacts only moderately with carboxyl groups [25]. The gel volume can therefore be assumed to contain Ca^2+^ cations capable of interacting with the introduced anionic RB molecules.

#### 2.1.3. Surface Area and Porosity of Xerogels(A) and Aerogels

**Figure 4:**
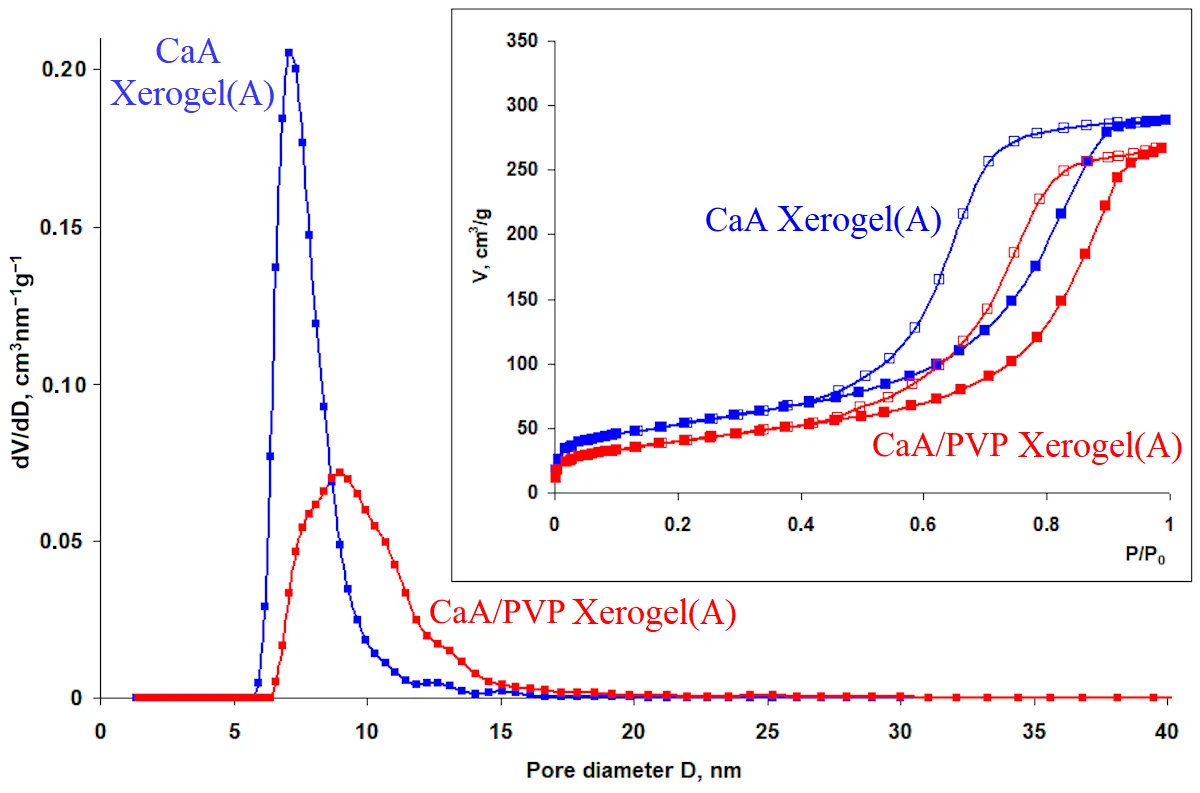
Pore-size distributions for the CaA xerogel(A) and CaA/PVP xerogel(A), with nitrogen (N_2_) adsorption (filled symbols) and desorption (empty symbols) isotherms at 77 K shown in the inset.

An unexpected result of this study was the mesoporosity found in the xerogel(A) samples formed by replacing the water in crosslinked CaA hydrogels with isopropyl alcohol and then air-drying them (see the Section 4). As shown in [13], the mesoporous structure of calcium alginate aerogels is characterized by a broad pore-size distribution, from 4–5 to 30–40 nm, and a specific surface area above 350 m^2^/g. At high relative pressures, the adsorption isotherm increased asymptotically toward 1, a behavior characteristic of samples containing macropores. The adsorption and desorption isotherms of CaA xerogels(A) and hybrid xerogels(A) (CaA + PVP) also exhibit hysteresis, indicating the mesoporous structure of these compounds. Unlike the aerogel isotherms, however, the xerogel(A) adsorption isotherms virtually reach a plateau at relative pressures P/P_0_ close to 1, indicating that all mesopores are filled with adsorbate and that no macropores are present in the samples. According to the DFT method, the pore-size distributions of the xerogels(A) are narrower, with maxima in the range 7–12 nm for different samples (Figure 4, Table 1).

Table 1 summarizes the measured surface areas (S_BET_), pore sizes, and total pore volumes of CaA and CaA/PVP xerogels(A), including RB-containing samples.

Incorporating the dye into the xerogels(A) and aerogels also decreases S_BET_ and the total pore volume (samples 3 and 5), while the mean pore size changes only slightly. This may be due to some rearrangement of the spatial structure of the carrier (decrease in porosity) during the immobilization process.

Introducing PVP into the xerogel(A) decreased both the specific surface area, S_BET_, and the total pore volume, V_t_, possibly because larger-diameter pores formed in the hybrid xerogel(A) than in the neat CaA xerogel(A) (Figure 4). It was previously shown that introducing polyvinylpyrrolidone into CaA aerogels increased the specific surface area to above 400 m^2^/g and increased the total gel pore volume. This effect was attributed to the formation of additional structures through interactions between the carboxyl moieties of calcium alginate and the nitrogen-containing groups of PVP in the three-dimensional gel network. The different effect of PVP on S_BET_ in an xerogel(A) may indicate the absence or disruption of specific CaA–PVP bonds after air-drying, whereas drying the gel specifically in supercritical CO_2_ may preserve such bonds. Nevertheless, the observed pore broadening in the hybrid CaA/PVP xerogel(A) may indicate that PVP retains some degree of association with the matrix.

Introducing the dye into hybrid CaA/PVP gels produces a marked increase in their total pore volume, probably because the CaA–PVP–RB complex system undergoes a rearrangement that evidently occurs before the gels are dried. In the resulting three-component CaA–PVP–RB system, the attenuation of the dye-induced decrease in V_t_ observed for the two-component CaA–RB system may indicate that the dye binds preferentially not to alginate moieties but to associated CaA–PVP groups. As shown below, this may decrease the dye-release rate constant for hybrid CaA/PVP xerogels(A) and aerogels relative to the corresponding CaA gels.

It should also be added that the de Boer t-plot method indicated the presence of a small number of micropores in the RB- and PVP-containing CaA xerogel(A) samples (samples 3 and 4). Their volume, however, was less than 1% of the total pore volume and thus lay within the range of statistical error.

#### 2.1.4. SEM and AFM Analysis of the Surface Structure

**Figure 5:**
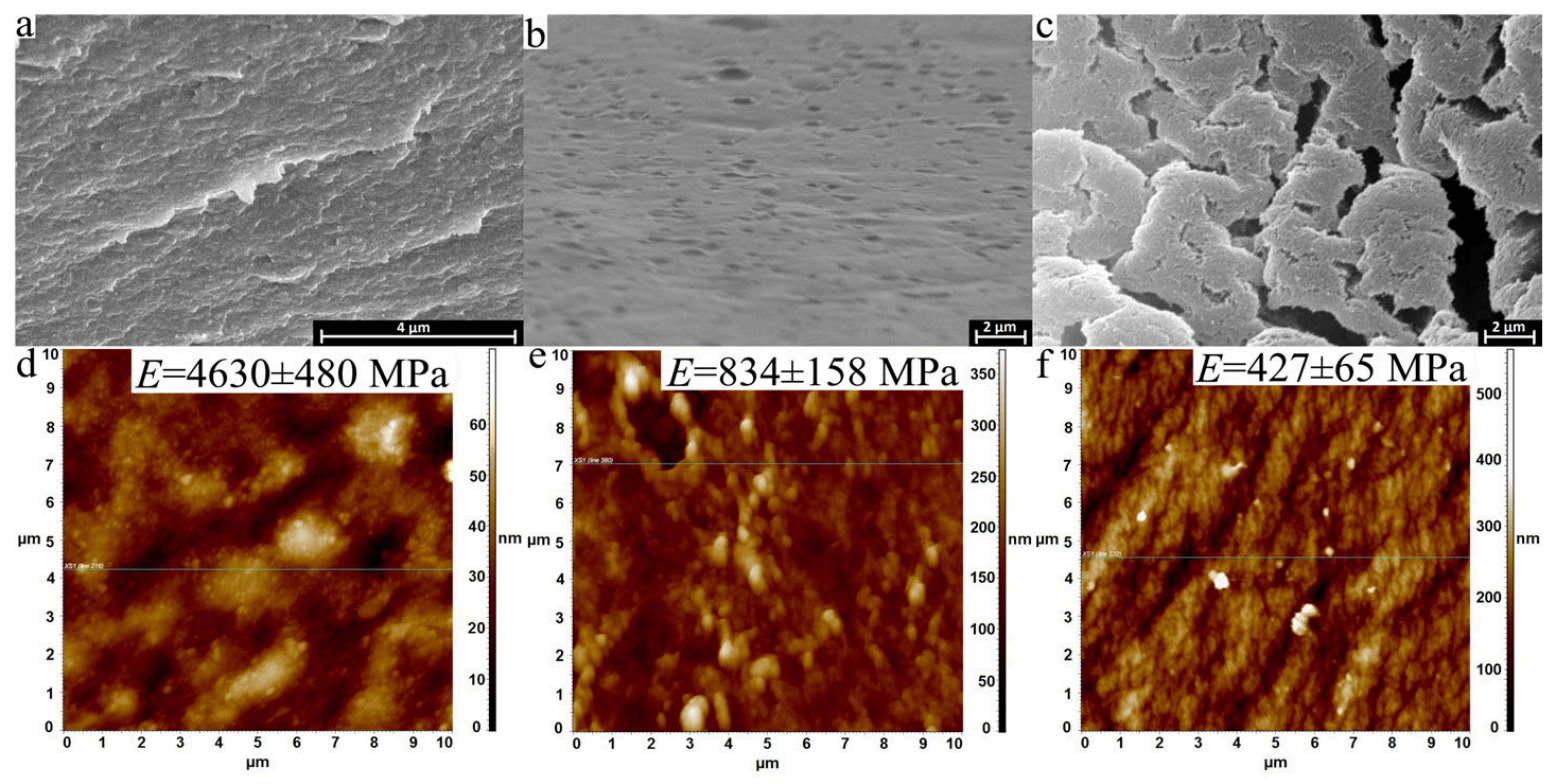
Structural and nanomechanical characterization of CaA gels: (**a**–**c**) SEM images of cross-sectional fracture surfaces of xerogel, xerogel(A) and aerogel films, respectively; (**d**–**f**) AFM images of 10 × 10 μm surface regions of xerogel, xerogel(A) and aerogel, respectively; with inset of local Young’s moduli (E) values. The xerogel images (**a**,**d**) are taken from [14].

The presence of a macroporous structure in the xerogels(A) and aerogels was demonstrated by SEM and confirmed by atomic force microscopy (Figure 5), indicating that these materials have a hierarchical structure. The SEM images show 13 × 20 μm regions of cross-sectional fracture surfaces of xerogel and aerogel films. Macropores ranging in width from hundreds of nanometers to several micrometers can be observed in the xerogels(A) and aerogels. All gel types exhibit a complex surface topography formed during gelation, i.e., during the interaction of sodium alginate with calcium salts.

The AFM images show progressively greater topographic variation from the xerogel to the xerogel(A) and aerogel. No distinct pores are visible in the xerogel image, whereas the xerogel(A) and aerogel surfaces contain depressions and more heterogeneous features. The mean profile height increased from 28.2 ± 0.9 nm for the xerogel to 168.5 ± 7.2 nm for the xerogel(A) and 260.4 ± 8.3 nm for the aerogel. The local Young’s modulus (E) decreased from 0.94 GPa for the xerogel to 0.43 GPa for the xerogel(A) and 0.33 GPa for the aerogel, consistent with lower local rigidity after solvent exchange and supercritical drying.

Flicker-noise spectroscopy (FNS) was used to parameterize the chaotic components of the AFM height profiles [26]; the methodological basis is described in the Supplementary Material. The spikiness factor S(L_01_^−1^) characterizes the contribution of spike-like irregularities to the power spectrum; L_01_ is their correlation length and n characterizes the rate of correlation loss over that interval. The parameter σ is the root-mean-square deviation associated with jump-like irregularities relative to the slowly varying baseline profile; L_1_ is the correlation length for jump-like height variations, and the Hurst exponent H_1_ characterizes the loss of memory over distances shorter than L_1_.

Low-frequency, resonant changes in surface topography were most pronounced for the xerogel over spatial scales from approximately 1.2 to 0.2 μm. Resonant variations were also observed for the aerogel, but over a narrower range, approximately 1.0–0.4 μm. Their contribution was less pronounced for the xerogel(A). The chaotic FNS parameters are summarized in Table 2.

The larger σ and S_c_(L_01_^−1^) values for the xerogel(A) and aerogel reflect greater jump- and spike-like surface irregularity than in the xerogel. The longer L_1_ and L_0_ values of the xerogel are consistent with a more spatially correlated surface.

### 2.2. Rose Bengal Immobilization in and Release from Calcium Alginate Gels

As noted above, RB can be introduced into calcium alginate gels either during the crosslinking of sodium alginate with calcium chloride or by additionally impregnating the gel with an isopropanol solution while the solvent in the gel pores is being replaced.

#### 2.2.1. Xerogels

**Figure 6:**
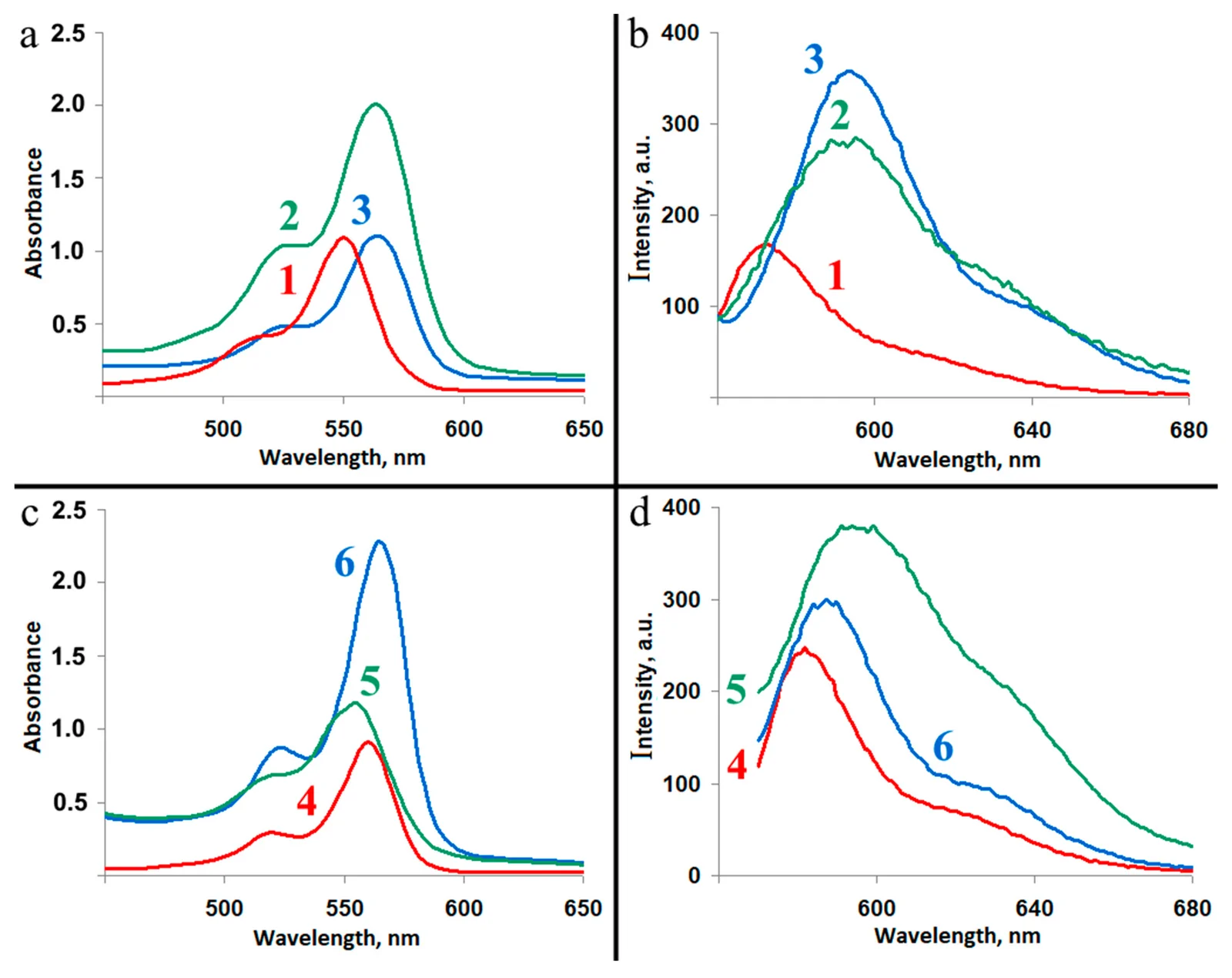
Electronic absorption (**a**,**c**) and fluorescence emission (**b**,**d**) spectra of RB. (**a**,**b**): RB in water (1), CaA xerogel (2), and CaA/PVP xerogel (3). (**c**,**d**): RB in isopropanol (4), CaA xerogel(A) (5), and CaA/PVP xerogel(A) (6).

RB incorporated into CaA xerogels and hybrid CaA/PVP xerogels, obtained from water, was not released from the matrix into distilled water, PBS, or isopropanol. Furthermore, RB did not diffuse into a preformed CaA hydrogel when the crosslinked gel was kept in an aqueous dye solution. Anionic RB may bind to calcium ions present throughout the xerogel. The electronic-absorption and fluorescence spectra also indicate that RB is present in an aggregated state in the CaA xerogel (Figure 6 and Table 3).

The presented spectral characteristics of RB in xerogel and aerogel matrices demonstrate bathochromic shifts both in the absorption spectra (Figure 6a,c) and in the fluorescence spectra (Figure 6b,d), which is obviously associated with a change in the molecular environment of the dye.

In particular, relative to the absorption spectrum of RB in water (Figure 6, curve 1), the spectrum of RB in the xerogel (curve 2) exhibits a bathochromic shift of approximately 15 nm. Such shifts may arise from dye aggregation and/or changes in the molecular environment of the chromophore [27,28]. The band near λ = 560 nm is assigned mainly to monomeric Rose Bengal, whereas the band near λ = 520 nm is associated with the dimeric form [28]. The D_520_/D_560_ ratio therefore indicates a greater relative dimer contribution in the CaA xerogel than in water, consistent with aggregation within the polyanion matrix [29]. In the hybrid CaA/PVP xerogel, the ratio shifts back toward the value observed for monomeric RB in water, indicating that PVP favors a less aggregated dye population. A 22 nm bathochromic shift in the emission maximum in the CaA xerogel likewise supports a change in RB aggregation and microenvironment [28].

**Figure 7:**
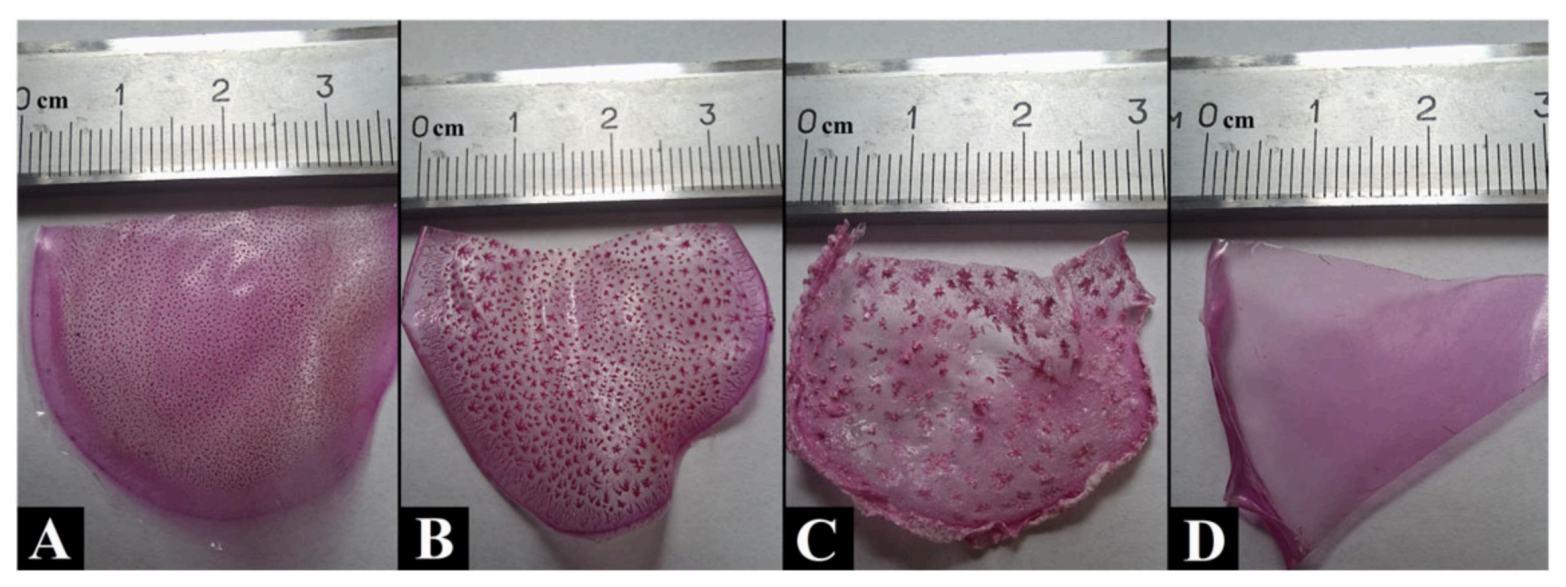
Photographs of Rose Bengal-loaded calcium alginate xerogels prepared with different calcium contents in the crosslinked gels: (**A**)—0.6, (**B**)—1.2, (**C**)—4.3, (**D**)—7.9 wt%. The Rose Bengal concentration was maintained at 0.5 wt% in all samples.

These conclusions are indirectly supported by the appearance of the Rose Bengal-containing xerogel films obtained at different calcium crosslinking cation contents. With an increasing Ca^2+^ content, the dye aggregates become larger and are presumably located near calcium ions (Figure 7). Panel D corresponds to xerogel samples with the maximum achievable degree of crosslinking, which were used in the release and activity studies.

#### 2.2.2. Xerogels(A)

The matrix also affects the spectral characteristics of the dye when RB is introduced into a CaA xerogel(A). Figure 6 compares the absorption and fluorescence spectra of RB in isopropanol (curve 4), in a calcium alginate xerogel(A) (curve 5), and in a hybrid CaA/PVP xerogel(A) (curve 6).

In the CaA xerogel(A) matrix, the RB fluorescence band undergoes a small bathochromic shift of 9 nm relative to the spectrum in isopropanol. The D_520_/D_560_ ratio indicates a greater aggregated-dye contribution in the CaA xerogel(A) than in the alcohol solution. In the hybrid CaA/PVP xerogel(A), however, the ratio decreases to 0.29, indicating disaggregation and a predominantly monomeric RB population. The fluorescence spectra support this interpretation.

#### 2.2.3. Release Kinetics in Phosphate-Buffered Saline

**Figure 8:**
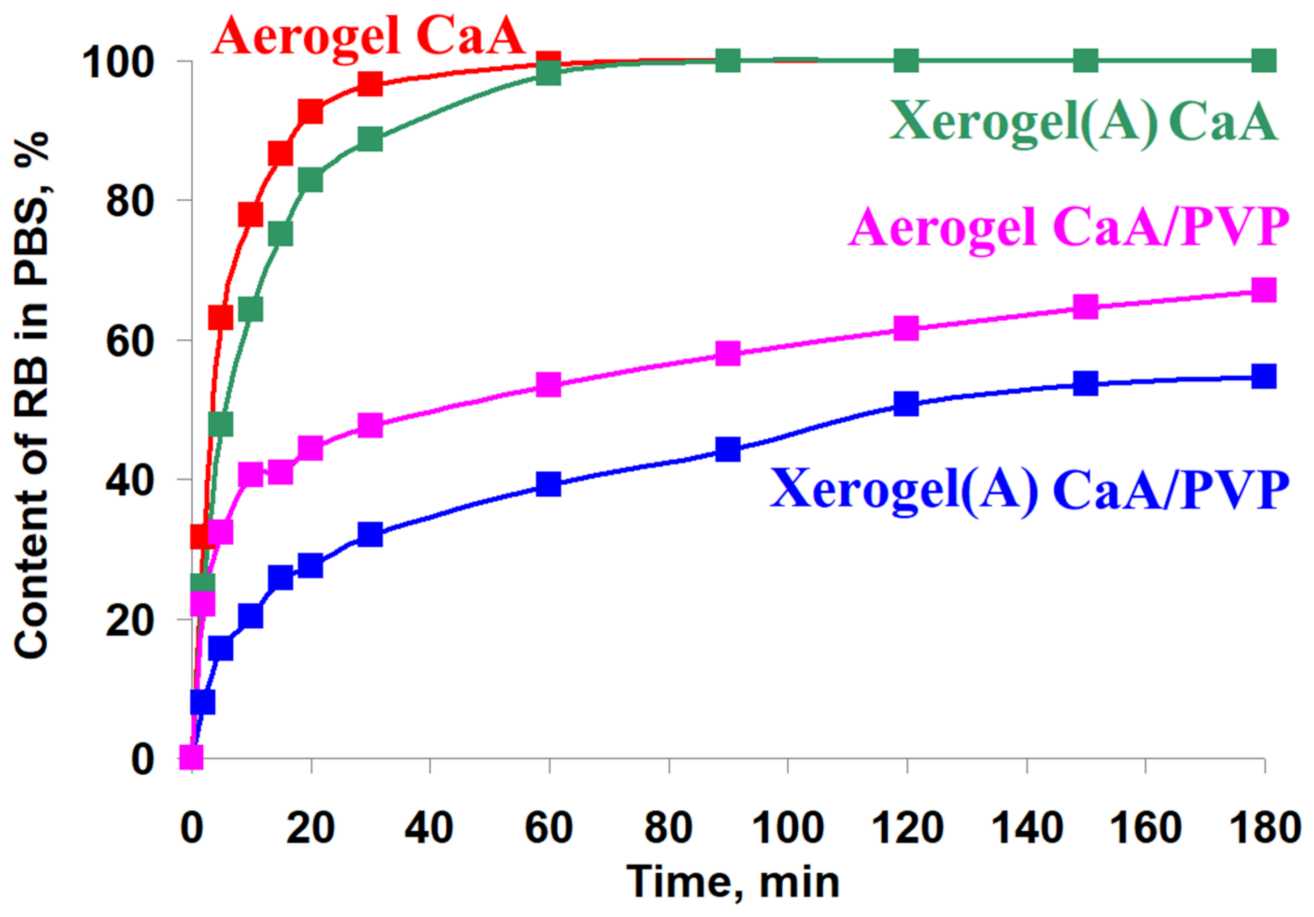
Release profiles of RB from CaA and CaA/PVP xerogel(A) and aerogel films into PBS (pH 7.2) at room temperature. Lines connect the experimental points as visual guides.

As noted above, RB incorporated into xerogel matrices, obtained from water, was not released into the surrounding media. The release kinetics from xerogels(A), obtained from isopropanol, and aerogels into PBS are shown in Figure 8 and summarized in Table 4.

PVP markedly decreases the rate of RB release from both xerogels(A) and aerogels. The release rate increases in the order CaA/PVP xerogel(A) < CaA/PVP aerogel < CaA xerogel(A) < CaA aerogel.

We previously observed faster diffusion of methylene blue from hybrid CaA/PVP gels than from CaA gels [14]. The contrasting behavior of anionic RB indicates that electrostatic interactions alone do not explain release from these matrices. PVP and chitosan are known to form complexes with molecular iodine and polyiodide species [30,31], while alginate/PVP blends can act as composite drug carriers [32]. These reports do not, however, demonstrate iodophor formation by the covalently iodinated RB molecule. Direct NOESY evidence has been reported for RB–PVP association [33]. Together with possible interactions of RB with Ca^2+^ and alginate, such an association may hinder release from xerogels and retard release from the more open xerogel(A) and aerogel networks.

### 2.3. Photosensitizing Activity of Rose Bengal Immobilized in CaA Gels in the Generation of ^1^O_2_

**Figure 9:**
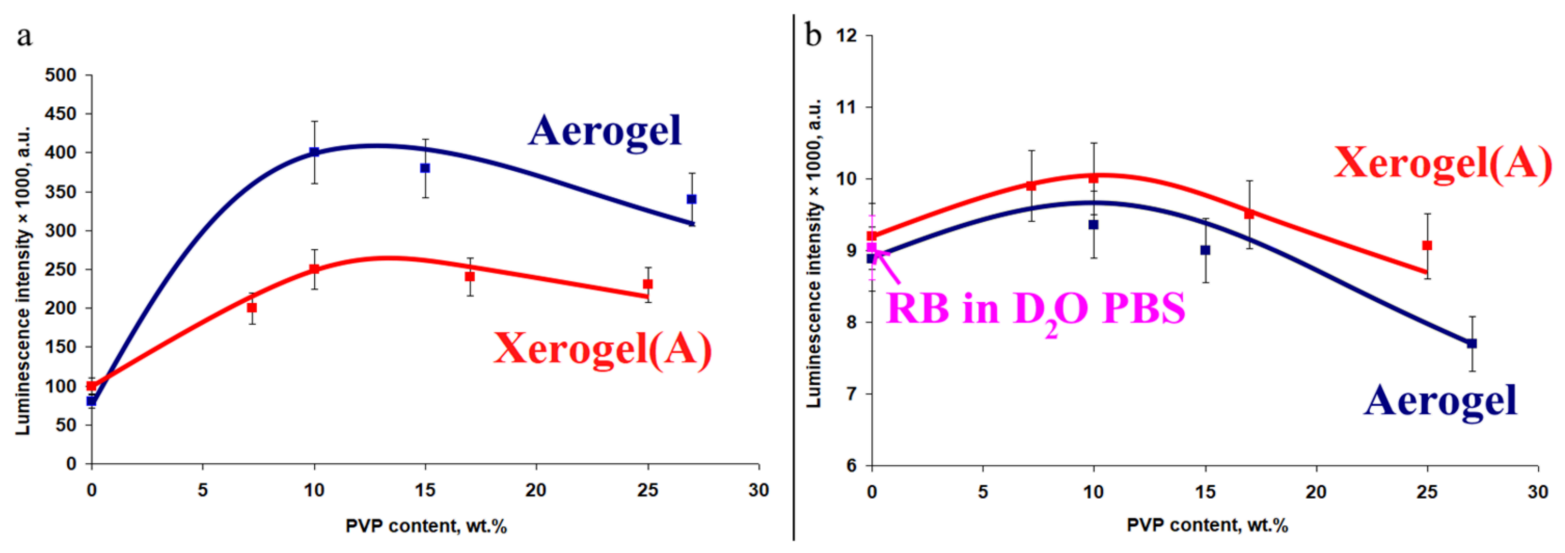
Dependence of singlet-oxygen luminescence intensity on PVP content for RB-containing CaA/PVP materials: (**a**) RB immobilized in aerogel and xerogel(A) films measured in air. RB concentration in films was 1wt%.; (**b**) RB released from xerogel(A) and aerogel films into PBS prepared with D_2_O. RB concentration in buffer was 5 × 10^−6^ M.

The singlet-oxygen-generation activity of the dye incorporated into CaA gels was studied from the ^1^O_2_ luminescence following photoexcitation of RB in the gel in air and after release of the dye into PBS prepared with D_2_O (Figure 9).

RB incorporated into CaA xerogels, obtained from water, exhibited no detectable ^1^O_2_ photogeneration under the conditions used. For xerogels(A), obtained from isopropanol, and aerogels, the luminescence response depended on matrix composition, supramolecular structure, porosity, and PVP content. In air, the hybrid CaA/PVP aerogel series showed the highest response, with a maximum or plateau near 10–15 wt% PVP. After release into PBS prepared with D_2_O, the xerogel(A) series produced the higher response. Previous studies showed that dye performance in ^1^O_2_ photogeneration depends on photosensitizer aggregation [34,35]. It was previously shown that PVP is capable of deaggregating BR, while the efficiency of the dye as a photosensitizer for ^1^O_2_ generation increased [36]. The absorption spectra of RB in hybrid matrices show an increased fraction of monomeric RB in the presence of PVP, consistent with the increased photochemical response. The lower local Young’s modulus and more open pore structure of the aerogels may additionally reduce matrix-induced restriction of immobilized RB.

These results demonstrate that Rose Bengal incorporated into xerogel (A) and aerogel films was reversibly bound to the alginate matrix, released into buffer solution (which mimics the cellular environment) at a sufficient rate, and showed high efficiency in singlet oxygen photogeneration. These findings suggest that such systems hold promise for applications in photodynamic therapy for superficial skin injuries. To further explore this potential, we plan to perform a series of in vitro studies. Specifically, we will evaluate their biocompatibility, toxicity, cell tropism, and photodynamic activity against the most relevant bacterial strains (*Escherichia coli and Staphylococcus aureus E. coli* and *S. aureus*).

## 3. Conclusions

A comparison of the physicochemical characteristics of calcium alginate-based xerogels and aerogels and their CaA/PVP hybrid analogs as carriers for the anionic photosensitizer “Rose Bengal,” RB, is presented. It is shown that the functional characteristics of the resulting gels—porosity, RB release, and its photoactivity in generating ^1^O_2_—depend on the drying method and matrix composition. In particular, it was established for the first time that CaA (A) xerogels obtained by replacing the solvent with isopropanol, followed by air-drying, form mesoporous systems with a BET surface area of 225 m^2^/g and an average pore diameter of 9.3 nm. It is also shown that RB, introduced from an aqueous solution during xerogel formation, is retained in the matrix in an aggregated state and is not released in PBS. However, RB incorporated into xerogels (A) and aerogels by treatment with isopropanol was retained less readily and was released into the environment. The release rate increased in the order CaA/PVP xerogel (A) > CaA/PVP aerogel > CaA xerogel (A) > CaA aerogel. Hybrid CaA/PVP aerogels demonstrated the highest activity in generating singlet oxygen in air, as measured by solid-state luminescence. Xerogels (A) are also attractive candidates for RB carriers for photodynamic therapy (PDT) applications, as they are prepared using a simplified procedure (without supercritical drying in the aerogel preparation method).

## 4. Materials and Methods

**Figure 10:**
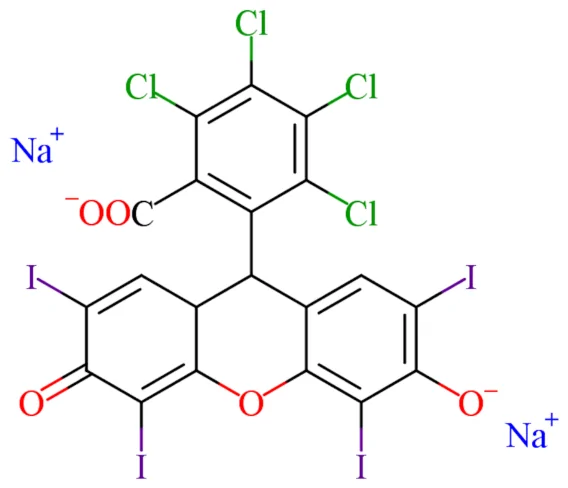
Chemical structure of Rose Bengal.

Sodium alginate (SA; Ruskhim; Moscow, Russia, Mw = 100–300 kDa), calcium chloride (granular, pure grade; Ruskhim, Moscow, Russia), Rose Bengal (RB, Figure 10) (4,5,6,7-tetrachloro-2′,4′,5′,7′-tetraiodofluorescein sodium salt; 95%, Sigma-Aldrich, Burlington, MA, USA), and polyvinylpyrrolidone (PVP, Sigma-Aldrich, St. Louis, MO, USA, 40 kDa) were used without further purification.

### 4.1. Preparation of Solid Alginate Gels

To obtain solid alginate gels as films, a 2 wt% aqueous sodium alginate solution was treated with a 5 wt% aqueous calcium chloride solution, kept for one day until a crosslinked hydrogel formed, and then washed with water. To evaluate the effect of the crosslinker content in the gel on Rose Bengal localization, calcium chloride solutions with mass concentrations ranging from 0.05 to 5 wt% were employed.

Xerogels were obtained in two ways:

- The hydrogels were air-dried for 7 h at 50 °C until a constant mass was reached (xerogels).
- The water in the hydrogel pores was gradually replaced with isopropyl alcohol over 4 days, producing xerogels. The xerogels were then air-dried at 50 °C for 4 h to a constant mass (xerogels(A)).

Calcium alginate aerogels were obtained by drying the corresponding alcogels in supercritical carbon dioxide (50 °C, 130 bar, 6 h). The solid-gel films were 100–150 μm thick.

Hybrid solid gels based on calcium alginate and additionally containing PVP were prepared in the same manner. PVP was introduced before gelation by mixing it with the sodium alginate solution at a mass ratio of 1:1. Adding PVP increased the thickness of the solid-gel films by a factor of approximately three. The PVP content, determined from the nitrogen content by elemental analysis, was up to 27 wt%. Nitrogen was measured using a PerkinElmer 2400 CHN analyzer (Norwalk, CT, USA), with a stated determination error of 0.3%.

The dye was immobilized in xerogels by introducing Rose Bengal into a sodium alginate solution or a combined SA/PVP solution (C_RB_ = 1 × 10^−5^ mol/L) before treatment with the crosslinking agent; the procedure described above was then followed. To obtain RB-containing calcium alginate xerogels(A) and aerogels, crosslinked CaA/RB hydrogels (or CaA/PVP/RB hydrogels) were kept for 4 days in isopropyl alcohol or in a solution of RB in isopropyl alcohol (C_RB_ = 1 × 10^−5^ mol/L), with periodic replacement of the alcoholic solution, and then dried in air (xerogel(A)) or in supercritical CO_2_ (aerogel). The Rose Bengal content of the solid gels was up to 1.5 wt%; this value was checked by completely extracting RB from the samples into PBS and determining its concentration spectrophotometrically, as described below.

### 4.2. Surface Area of the Solid Gels

The specific surface area and porous-structure parameters of the samples were determined by low-temperature nitrogen sorption (77.4 K) using a NOVA 1200e gas-sorption analyzer (Quantachrome Instruments, Boynton Beach, FL, USA). High-purity nitrogen gas was used as the adsorbate. Before measurement, the samples were degassed under vacuum at 50 °C for 3 h. The specific surface area was determined by the Brunauer–Emmett–Teller (BET) method over a relative-pressure range of P/P_0_ = 0.05–0.21, where P_0_ is the saturated vapor pressure of the adsorbate at the experimental temperature. The experiments were repeated three times (n = 3). Table 1 presents the average values. Pore-size distributions were determined by quenched solid density functional theory (QSDFT) [37,38] using a cylindrical-pore model.

### 4.3. Surface Structure of Crosslinked Alginate-Gel Films and Fracture Surfaces

The surface topography of the solid gels was examined using an atomic force microscope (NT-MDT, Zelenograd, Russia) with Etalon probes (NT-MDT, Russia). For each sample, 5–7 images measuring 10 × 10 μm^2^ were analyzed in semicontact mode. The local Young’s modulus of the aerogels was measured with a BioScope Resolve atomic force microscope (Bruker, Billerica, MA, USA) in Force Volume Mapping mode. An RTESPA-150 probe with calibrated cantilever spring constant k_r_ = 2.258 N/m and tip radius R_T_ = 18.7 nm was used. Local-stiffness maps measuring 80 × 80 μm^2^, at a resolution of 40 × 40 points, were obtained. The results were averaged over 5–6 images for each sample, and the maps were processed using NanoScope Analysis 1.8.

The structure of cross-sectional fracture surfaces of the solid-gel films was examined by scanning electron microscopy using a Melytec SV 32 microscope (Moscow, Russia) under high vacuum at an accelerating voltage of 15–20 kV.

### 4.4. IR Spectroscopy

IR spectra of the initial reagents and the resulting systems were recorded using a Spectrum Two FT-IR spectrometer (PerkinElmer) in attenuated total reflectance (ATR) mode. A high-performance LiTaO_3_ IR detector operated at room temperature, and a standard optical arrangement with KBr windows was used. Data were collected over the wavenumber range 4000–450 cm^−1^ at a resolution of 0.5 cm^−1^. The spectral data were converted to IR transmittance. The spectra were normalized using the asymmetric stretching band of the carboxylate COO^−^ groups at 1600 cm^−1^ as an internal standard.

### 4.5. Differential Thermal Analysis

Differential thermal analysis (DTA) was used to determine the presence of bound water in the structure of the solid gels. Measurements were performed using an STA 449 F3 simultaneous thermal analyzer (NETZSCH, Zelb, Germany). Sample masses were 10–12 mg. The polymers were thermally degraded in air at a gas flow rate of 20 mL/min and a linear heating rate of 10 °C/min.

Sample mass loss was recorded to within 10^−3^ mg; the relative measurement errors were ±1.5 °C for temperature and ±3% for thermal effects. The degradation process was described in terms of mass loss (TG), mass-loss rate (DTG), and thermal effects (DSC) as functions of temperature. The following parameters were determined: onset temperature of mass loss (T_bmi_), onset temperature of oxidation (T_oxb_), total thermal effect of the process (Q, kJ/g), and maximum mass-loss rate (W_max_).

### 4.6. Kinetics of Rose Bengal Release from Solid Gels into the External Medium

The diffusion of RB from impregnated CaA xerogels(A) and aerogels was studied at room temperature in phosphate-buffered saline (PBS; Eco-Service, Russia; pH~7.2), which simulated the biological cellular environment. An RB-containing CaA xerogel(A) or aerogel polymer film (mass ~0.010 g) was placed in 10 mL of buffer. The solution was stirred at room temperature with a magnetic stirrer at 200 rpm until the dye had been completely released into the buffer (0.5–24 h). To determine the amount of dye released from the polymer matrix, the optical density of the solution, D, was measured at fixed time intervals (λ_RB_ = 550 nm). Absorption spectra were recorded with a Cary 50 spectrophotometer (Varian, Palo Alto, CA, USA). The experiments were repeated three times (n = 3). Table 4 presents the average values. The measurement error did not exceed 5%.

The optical-density measurements for the PBS solution containing dye released from the polymer matrices were represented as the dependence of the Rose Bengal concentration in PBS, C(t), on time t. The total amount of RB introduced into the polymer matrix, C_tot_, was determined spectrophotometrically by measuring its concentration in PBS after complete dye release. All dependences at the initial stage of dye release were described by a first-order kinetic equation with an accuracy above 95%:

(1) C(t) = C_tot_ × [1 − exp(−kt)],

At the initial stage of RB release from the matrix into the buffer, when C(t) << C_tot_ the equation becomes the following:

(2) C(t)/C_tot_ ≈ k × t,

Equation (2) was therefore used to determine the rate constants (k) for RB release from xerogel(A)- and aerogel-based CaA polymer matrices during the initial release stage, i.e., over the first linear segment of the kinetic curve. The kinetic curve obtained using Equation (2) was also used to determine the times required to release 50% and 100% of the RB from the polymer matrices into PBS.

In all cases considered in this study, the observed deviations from first-order kinetics resulted in errors of no more than 5% in the calculated rate constants, k.

### 4.7. RB Activity in the Photogeneration of Singlet Oxygen

The activity of RB introduced into alginate xerogels(A) or aerogels (samples with linear dimensions of 2 cm × 1 cm) in the photogeneration of singlet oxygen ^1^O_2_ in air and in a phosphate buffer was determined by the intensity of ^1^O_2_ luminescence in the near-infrared region (*λ* = 1267 nm). Luminescence spectra was recorded using a Horiba Fluoromax Plus spectrofluorimeter (Irvine, CA, USA) with a DSS-IGA020L detector. Irradiation power—50 mW, irradiation time 100 s at room temperature. To determine the RB activity during the ^1^O_2_ generation in air, samples with an RB content of 1.0–1.5 wt% and a PVP content in hybrid gels up to 27 wt% were excited by light with a wavelength of 560 nm. PVP increases the thickness of the xerogel (A) films. Before measuring singlet oxygen luminescence in the alginate films containing the dye, the signal at 1267 nm was recorded for the corresponding blank films based on CaA and CaA + PVP and subsequently subtracted. The ^1^O_2_ luminescence intensity was calculated as the average value for 10 measurements for each sample; the measurement error was 10%.

To determine the activity of RB during the generation of ^1^O_2_ PBS in deuterated water, the samples were placed in a cuvette with a freshly prepared buffer of 3 mL. The solution was intensively stirred with a magnetic stirrer. When the concentration of RB in solution reached 5 × 10^−6^ M (monitored by the optical density of the RB absorption band with *λ* = 560 nm) (approximately 2–5 min), the aerogel film was removed. The resulting solution containing 5 × 10^−6^ M of RB was excited by light with *λ* = 550 nm, and the luminescence intensity of ^1^O_2_ was recorded at *λ* = 1267 nm. The luminescence intensity of ^1^O_2_ was calculated as the average value for 3 measurements; the measurement error was 5%. For comparative evaluation, a freshly prepared RB solution at a concentration of 5 × 10^−6^ M in deuterated PBS buffer was used as a standard; the ^1^O_2_ luminescence intensity of this standard was 0.0090 ± 0.0005 a.u.

## Figures and Tables

**Table 1:** Specific surface area and porous-structure parameters of calcium-alginate-based xerogels(A) and aerogels.

№	GelType	Composition	S_BET_^a^, m^2^/g	V_t_^b^, cm^3^/g	d_mp_^c^, nm	d_DFT_^d^, nm	V_DFT_^d^, cm^3^/g	S_DFT_^d^, m^2^/g
1	Xerogel(A)	CaA	225	0.520	9.3	7.1	0.50	210
2	Xerogel(A)	CaA/PVP *	129	0.366	11.4	9.0	0.35	121
3	Xerogel(A)	CaA/RB	123	0.266	8.6	7.3	0.26	119
4	Xerogel(A)	CaA/PVP */RB	160	0.556	13.9	12.7	0.54	143
5	Aerogel	CaA/RB	302	1.94	25.7	24.8	1.81	300
6	Aerogel	CaA/PVP */RB	243	2.12	32.1	31.5	1.86	240

The error in determining SBET did not exceed 10%. * PVP content: 10 wt%; RB content: 1.5 wt%. ^a^ Specific surface area determined by the BET method. ^b^ Total pore volume at P/P_0_ = 0.99. ^c^ Mean pore diameter, d_mp_ = 4 V_t_/S_BET_. ^d^ Pore diameter, volume, and surface area determined by the DFT method.

**Table 2:** Chaotic FNS parameters of the alginate-gel surfaces.

Gel Type	<h>, nm	σ, nm	S_c_(L_01_^−1^), nm^2^·*f*_d_^−1^	L_1_, μm	L_01_, μm	n	H_1_
Xerogel	28.2 ± 0.9	5.2 ± 1.7	1.7 ± 0.7	0.9 ± 0.04	0.7 ± 0.07	2.2 ± 0.1	0.8 ± 0.2
Xerogel(A)	168.5 ± 7.2	35.3 ± 4.1	18.7 ± 7.2	0.4 ± 0.1	0.3 ± 0.06	2.7 ± 0.2	0.9 ± 0.2
Aerogel	260.4 ± 8.3	47.9 ± 2.6	36.3 ± 7.0	0.3 ± 0.05	0.3 ± 0.09	2.5 ± 0.1	1.0 ± 0.1

*f*_d_—spatial sampling frequency = 51 μm^−1^.

**Table 3:** Spectral parameters of RB in solutions and calcium alginate matrices.

№	Samples	Absorbance λ_max_, nm	D_520_/D_560_ (Dimer/Monomer)	Emission λ_lum_, nm
1	RB in water	550	0.32	573
2	RB in CaA xerogel	565	0.46	595
3	RB in CaA/PVP xerogel	565	0.30	593
4	RB in iPrOH	560	0.32	582
5	RB in CaA xerogel(A)	555	0.58	591
6	RB in CaA/PVP xerogel(A)	565	0.29	587

**Table 4:** Kinetic parameters reported for RB release from CaA- and CaA/PVP-based xerogels(A) and aerogels into PBS.

Samples	k × 10^2^, s^−1^	Time to 50% RB Diffusion, min	Time to 100% RB Diffusion, min
CaA xerogel(A)	8.1 ± 0.4	5	60
CaA/PVP xerogel(A)	4.9 ± 0.3	120	640
CaA aerogel	9.1 ± 0.6	5	34
CaA/PVP aerogel	6.5 ± 0.4	50	240

## Data Availability

The data presented in this study are available upon request from the corresponding author.

## Supplementary Materials

The following supporting information can be downloaded at https://www.mdpi.com/article/10.3390/gels12080741/s1, The basis of the FNS approach.

## Abbreviations

The following abbreviations are used in this manuscript:

CaA	Calcium alginate
RB	Rose Bengal
PVP	Polyvinylpyrrolidone
PBS	Phosphate-buffered saline
PS	Photosensitizer
PDT	Photodynamic therapy
SA	Sodium alginate
DTA	Differential thermal analysis
DTG	Differential thermal gravimetry
DSC	Differential scanning calorimetry
DFT	Density functional theory
SEM	Scanning electron microscopy
AFM	Atomic force microscopy
FNS	Flicker-noise spectroscopy
NOESY	Nuclear overhauser effect spectroscopy
