# Supplementary material for: Rose Bengal Immobilization in and Release from Calcium Alginate Gels for Singlet-Oxygen-Generating Photosensitizing Systems
Source: Gels. 2026 Aug 19;12(8):741. doi: 10.3390/gels12080741 (PMC13512124; doi:10.3390/gels12080741)
Supplement: Supplementary file 1 [file gels-12-00741-s001.zip › gels-4495216-supplementary.pdf]

The Basis of the FNS Approach

The FNS approach to analyzing digitized data acquired by scanning the surface relief and measuring the sequence (spatial series) of surface-profile heights h(x) assigns information significance to the correlations that appear within the measured height sequence at different spatial shifts Δ. These correlations arise not only for the regular ("resonant") components of h(x), but also within sequences of signal irregularities ("jumps" and "spikes") at each spatiotemporal level of the hierarchy of the surface topography under study.

According to the FNS methodology, the information contained in the heights h(x) of each surface-relief profile is related to the dependence of the "subsequent" profile-height values h(x + Δ) (0 ≤ Δ ≤ L) on the "preceding" values. The local heights h(x) may therefore be treated as dynamic variables of the system under study, and the surface parameters obtained from each relief profile may be related to the autocorrelation function commonly used in statistical physics:

$$\psi(\tau) = \langle h(x)h(x+\Delta)\rangle_{L-\Delta}, \qquad \langle(\ldots)\rangle_{L-\Delta} = \frac{1}{L-\Delta}\int_0^{L-\Delta}(\ldots)dx,$$

which characterizes the relationship between the values of the dynamic variable h(x) at larger and smaller values of its argument.

To determine the introduced topographic parameters of the surfaces under study, each set of 512 lines forming an AFM image was divided into eight equal groups ("bands") of 64 lines: lines 1–64, 65–128, and so on. For each group, an "averaged" surface-relief profile $h_i(x)$ (i = 1, …, 8) was calculated; the index i denotes the group of scans counted from the bottom of the image. The FNS parameters were calculated for the complete set of these averaged surface-relief profiles. Among the parameters derived from the AFM images, the mean recorded "roughness height" ⟨h⟩ should be distinguished from parameters characterizing slowly varying, low-frequency regular ("resonant") changes in surface topography and from parameters characterizing high-frequency chaotic topographic variations. The latter quantify the intensity and correlation relationships of the irregularities—jumps and spikes—in the recorded profiles.
